# Endothelial mitochondrial homeostasis in the development and progression of atherosclerosis

**DOI:** 10.3389/fcvm.2026.1813122

**Published:** 2026-06-12

**Authors:** Naznin Sultana Remex, Md Sakil Miah, Tamjid Islam, Richa Aishwarya, Md Hasif Sinha, Proma Dhar, Cyrine Ben Dhaou, A. Wayne Orr, Md Shenuarin Bhuiyan

**Affiliations:** 1 Department of Molecular and Cellular Physiology, Louisiana State University Health, Shreveport, LA, United States; 2 Department of Pathology and Translational Pathobiology, Louisiana State University Health, Shreveport, LA, United States

**Keywords:** atherrosclerosis, endothelial dysfunction, endothelial cell, mitochondria, mitochondrial signaling pathway

## Abstract

Vascular endothelial cells (ECs) play a critical role in vascular functional homeostasis, and endothelial dysfunction activates signaling pathways that drive the development and progression of atherosclerosis. Mitochondria in ECs play an essential signaling role in regulating redox balance, calcium signaling, metabolic signaling, and inflammatory responses. Disruption of mitochondrial functional homeostasis by atherogenic stimuli leads to excessive mitochondrial reactive oxygen species production, altered mitochondrial dynamics, defective mitophagy, and mitochondrial DNA damage. These mitochondrial defects in ECs reduce nitric oxide bioavailability through eNOS uncoupling, destabilize endothelial junctional complexes, and promote endothelial activation. Additionally, damaged mitochondria release mitochondrial danger-associated molecular patterns contributing to the activation of inflammation and redox-sensitive signaling pathways in ECs. In this review, we delineated the mechanistic links between endothelial mitochondrial dysfunction and the pathological features of atherosclerosis. We highlight the contribution of mitochondrial signaling to the regulation of oxidative stress, innate immune activation, and endothelial barrier disruption. We also discussed emerging therapeutic strategies targeting mitochondria-associated signaling pathways, including modulation of mitochondrial dynamics, mitophagy, redox signaling, and mitochondria-targeted drug delivery. Together, we provide insights into the role of endothelial mitochondria in atherosclerotic disease progression and compelling targets for mechanism-based therapeutic intervention.

## Graphical Abstract

**Figure:**
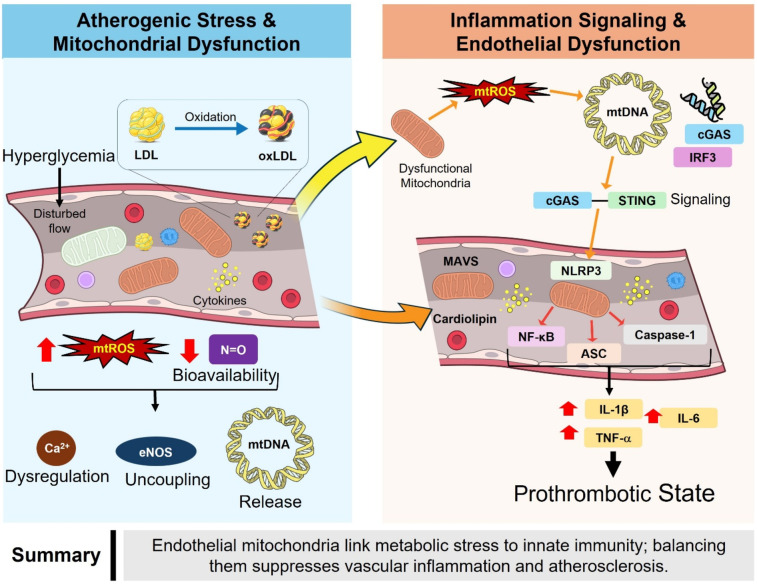

## 1 Introduction

Cardiovascular diseases (CVDs) remain the leading cause of mortality and morbidity worldwide despite substantial advances in therapeutics to prevent and heal heart diseases (1–3). Approximately 18 million individuals die from CVDs annually, a number projected to rise to nearly 24 million by 2030. Although historically more prevalent in high-income countries, recent studies suggest CVDs as the leading cause of death in low- and middle-income nations, underscoring their expanding global burden. CVDs arise from a multifactorial interplay of behavioral and metabolic risk factors, including unhealthy diet, physical inactivity, tobacco use, obesity, diabetes, aging, and genetic predisposition. Among CVDs, atherosclerosis is the predominant pathological process underlying coronary artery disease, stroke, and peripheral arterial disease (4).

### 1.1 Atherosclerotic cardiovascular disease

Atherosclerosis is a chronic inflammatory disease of the arterial wall characterized by lipid accumulation, plaque formation, luminal narrowing, and eventually plaque rupture that leads to acute thrombotic events. The cascade of events associated with the development of atherosclerosis includes activation of the endothelium, endothelial dysfunction, formation of early fatty streak and necrotic core, followed by more complex advanced plaque formation, fibrous cap formation, and plaque rupture (5). Build-up of plaque towards the arterial lumen leads to obstruction of blood flow, resulting in tissue ischemia (6). Rupture of an advanced plaque can cause life-threatening events like thrombosis or even death (7). In Western societies, atherosclerosis accounts for approximately half of all deaths, with prevalence increasing with age and following the loss of estrogen-mediated vascular protection in postmenopausal women.

### 1.2 Endothelial cells in atherosclerosis

The vascular endothelium is a functionally heterogeneous, metabolically active monolayer of endothelial cells (ECs) lining the luminal surface of all blood vessels. ECs exist in two primary states: a quiescent state that maintains vascular homeostasis in healthy adults and an activated state that responds to physiological needs or pathological stressors. ECs respond rapidly to environmental changes and transition between quiescent and activated states in response to stimuli such as hypoxia, ischemia, and inflammation. Dysfunction in ECs has been recognized as one of the earliest events in atherosclerosis development (8). Atherogenic stimuli activate ECs, resulting in the recruitment and accumulation of monocytes, which differentiate to macrophages and engulf lipids, becoming foam cells (9). As the disease progresses, endothelial dysfunction is accompanied by increased reactive oxygen species (ROS) generation at the expense of nitric oxide (NO) and hydrogen sulfide (H_2_S), thereby promoting vascular inflammation and sustained inflammatory cytokine production, accelerating plaque development (10, 11). Also, perturbed flow-dependent mechanisms promote atherogenesis.

### 1.3 Mitochondria in atherosclerotic plaque formation

Accumulating evidence indicates that mitochondrial dysfunction is a key upstream regulator of these pathological processes. Mitochondrial defects in bioenergetics, dynamics, calcium handling, and mitochondrial DNA (mtDNA) integrity led to excessive generation of ROS, establishing a self-amplifying cycle of oxidative injury and inflammation. These mitochondrial-derived ROS (mtROS) reduce NO bioavailability, impair endothelial NO synthase (eNOS) activity, and promote LDL oxidation, leading to endothelial activation and atheroma formation. Therefore, in this review article, we examined evidence for endothelial mitochondrial dysfunction as a central driver of vascular pathology in atherosclerosis and discussed mitochondrial pathways as emerging therapeutic targets.

## 2 Endothelial metabolism and mitochondria

ECs are dynamic entities that can undergo metabolic reprogramming in response to physiological and pathological conditions (12). This functional plasticity of ECs depends on metabolic flexibility, enabling dynamic reprogramming of glycolytic and mitochondrial pathways to support activities such as migration, proliferation, and differentiation (8, 13).

### 2.1 Endothelial cell metabolism (glycolysis, fatty acid oxidation, and amino acids)

Under physiological conditions, ECs produce 85% of ATP through anaerobic glycolysis in various EC types (14). For instance, rat coronary microvascular ECs convert ∼98% of glucose to lactate (15), pig aortic ECs generate more than 75% of cellular ATP through glycolysis (16), and human umbilical vein ECs (HUVECs) showed glycolytic flux > 200-fold higher than glucose oxidation fluxes (17). Comparative studies in pulmonary microvascular ECs (PMVECs; rapid growth and highly glycolytic) and arterial ECs (PAECs; slower growth and more oxidative) have shown twofold higher total cellular ATP content in PMVECs compared to PAECs (18). In healthy vasculature, the majority of ECs remain quiescent, having lower metabolic rates, express reduced metabolic gene transcripts, and utilize FAO to maintain redox homeostasis compared with highly activated ECs (19). Laminar shear stress maintains ECs in the quiescent state of ECs by inhibiting glycolysis, whereas angiogenic stimuli promotes EC proliferative state switching to glycolysis as a fuel source (20–22). ECs' unique metabolic adaptation and flexibility to switch between angiogenic and quiescent states are essential for sustaining proliferation, migration, and adaptive responses to environmental stressors (23). High rates of glycolysis in ECs facilitate the synthesis of macromolecules by channeling glucose into glycolytic pathways necessary for angiogenesis, proliferation, and migration. Angiogenesis activation increases glycolysis by twofold in ECs, enabling the acquisition of a proliferative, migratory, and morphogenic phenotype under conditions such as wound repair, physical activity, hormonal cycles, and cancer progression (13).

ECs generate very little ATP by oxidation of glucose, fatty acids (FA), and amino acids through the mitochondrial electron transport chain (ETC) (21). Under physiological conditions, only ∼1–3% of glucose enters the pentose phosphate pathway (PPP) (24). However, glucose flux can be substantially redirected into the PPP by up to ∼80% during oxidative stress, enhancing NADPH production, thereby maintaining reduced glutathione (GSH) levels, supporting antioxidant defense, and limiting ROS-mediated damage (15, 24, 25). A smaller fraction of glucose is also diverted into the hexosamine biosynthesis pathway, contributing to protein glycosylation. Accordingly, the vascular endothelium functions as a metabolically active organ with systemic influence on metabolic homeostasis (26). Several endothelial-expressed metabolic regulators have been implicated in regulating whole-body glucose metabolism (27, 28), including insulin receptor substrate-2 (IRS2) (29), peroxisome proliferator-activated receptor-*γ* (PPAR*γ*) (30), and FA translocase (FAT/CD36) (31).

ECs are capable of oxidizing both extracellular and intracellular FA, although the relative contribution of fatty acid oxidation (FAO) in comparison with glycolysis to ATP production remains debated (32). ECs also metabolize FAs to generate acetyl-CoA to fuel the tricarboxylic acid (TCA) cycle. FAO, along with anaplerotic substrates, supports deoxynucleotide triphosphate (dNTP) synthesis to support EC proliferation (33). Disruption of FA oxidation in ECs through genetic or pharmacological inhibition of carnitine palmitoyltransferase-1 (CPT1) impairs endothelial differentiation, proliferation, and barrier function (34, 35). Similarly, loss of FA-binding protein-4 (FABP4) reduces endothelial proliferation, migration, and sprouting, underscoring the importance of lipid-derived metabolic signaling in endothelial function (36).

**Figure 1:**
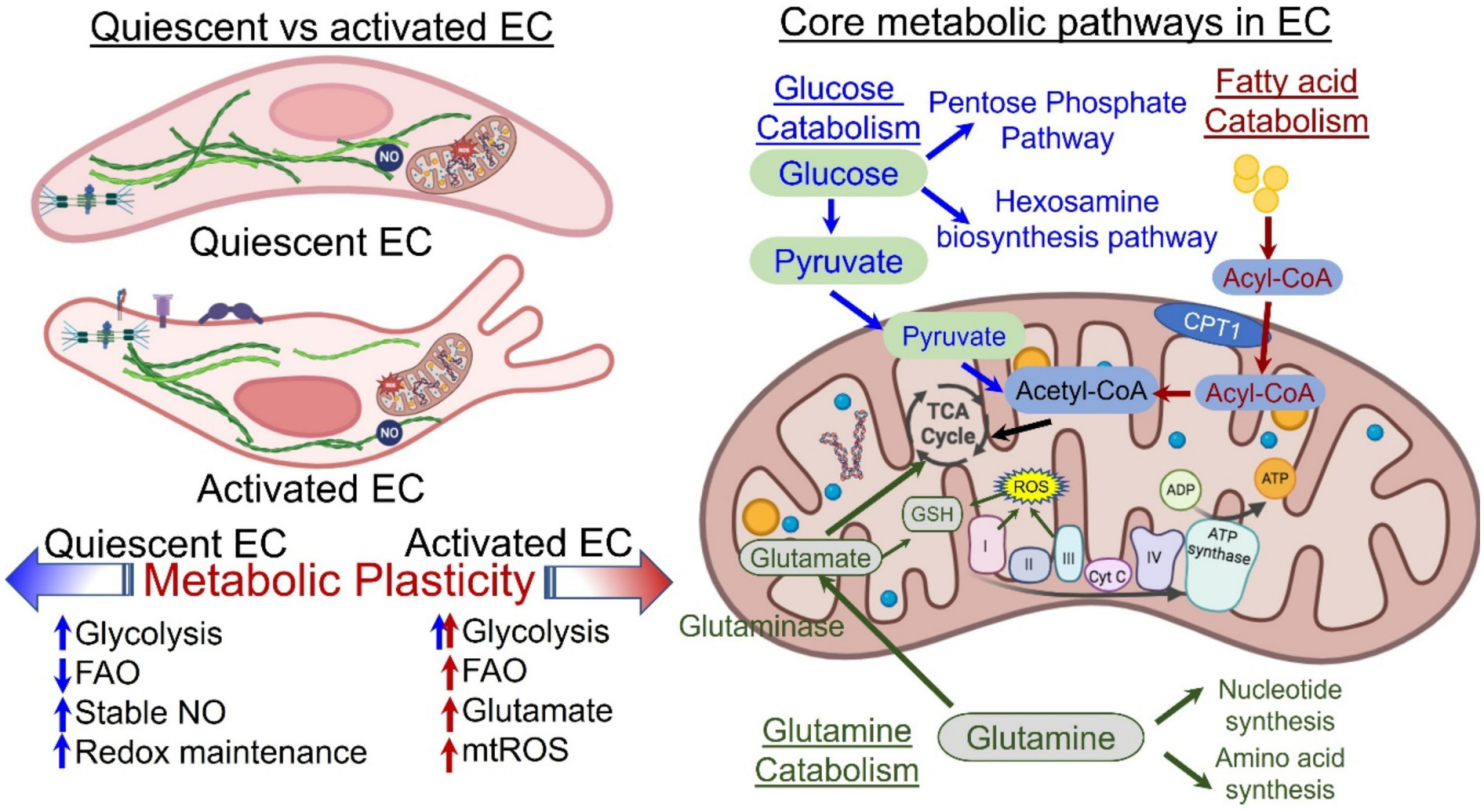
Metabolic plasticity and core metabolic pathways in ECs. Endothelial cells dynamically transition between quiescent and activated states in response to stimuli. Quiescent ECs are characterized by balanced highly dependent on glycolysis, stable NO production, and maintained redox homeostasis. In contrast, activated ECs exhibit metabolic reprogramming marked by increased glycolysis, enhanced fatty acid oxidation, and glutamine utilization, elevated mtROS, and altered NO signaling. Glucose is primarily metabolized through glycolysis to pyruvate, which can enter mitochondria to generate acetyl-CoA and fuel the TCA cycle. A portion of glucose is diverted into the pentose phosphate pathway (PPP) to generate NADPH for glutathione (GSH)-dependent antioxidant defense, or into the hexosamine biosynthesis pathway for protein glycosylation. Fatty acids undergo *β*-oxidation via CPT1-dependent mitochondrial import to produce acetyl-CoA, contributing to TCA cycle activity and biosynthetic processes. Glutamine is converted by glutaminase to glutamate, supporting TCA cycle anaplerosis, nucleotide synthesis, amino acid production, and redox balance. Mitochondrial oxidative phosphorylation generates ATP and reactive oxygen species (ROS), integrating metabolic and redox signaling in endothelial function.

Among amino acids, ECs can oxidize glutamine, glutamate, and alanine at substantial rates In glucose-deprived conditions (15). Glutamine is the most consumed amino acid by ECs and plays a critical role in sustaining proliferation and vascular expansion (37, 38). Circulating glutamine is abundant in human plasma, and ECs express glutaminase (GLS), which converts glutamine to glutamate (39). Glutamine also serves as a precursor for amino acids such as glutamate, asparagine, and aspartate, and contributes to the TCA cycle anaplerosis and maintenance of redox homeostasis via GSH synthesis (40). Approximately 30% of TCA cycle carbon in ECs is derived from glutamine, a contribution comparable to that of glycolysis and FAO (33). Moreover, glutamine-derived glutathione is essential for redox homeostasis, and glutamine depletion increases EC susceptibility to ROS-induced injury (37). Overall, metabolic flexibility of ECs allows to dynamically switch among glycolysis, FA oxidation, and glutaminolysis enables vascular cells to adapt to functional demands, including angiogenesis, wound healing, and environmental stress, thereby preserving vascular homeostasis (29–31, 41, 42) (Figure 1).

### 2.2 Endothelial mitochondria: specialized structure and homeostatic functions

Mitochondria in ECs are structurally conserved but functionally distinct from those in highly oxidative tissues such as cardiomyocytes. Unlike oxidative tissues, which depend heavily on mitochondrial oxidative phosphorylation for ATP production, ECs rely predominantly on glycolysis to meet their energy demands (43). Consequently, mitochondrial content in ECs is relatively low, accounting for approximately 2–6% of total cytoplasmic volume, compared with ∼30–35% in cardiomyocytes (27, 28). This limited mitochondrial abundance in ECs reflects a functional specialization toward signaling and regulatory roles in the vascular endothelium.

Pathological stimuli such as ischemia/reperfusion (I/R) injury and hyperglycemia impose metabolic stress on endothelial mitochondria by increasing the supply of reducing equivalents (NADH and FADH₂), leading to mitochondrial membrane hyperpolarization. This excessive hyperpolarization impedes forward electron transfer through the ETC, promoting electron leak and the partial reduction of oxygen to generate mitochondrial ROS (44–46). Under hypoxic conditions, endothelial mitochondria undergo microtubule-dependent perinuclear clustering, positioning ROS production proximal to the nucleus. This spatial reorganization facilitates redox-dependent regulation of hypoxia-responsive gene expression, thereby contributing to endothelial dysfunction (47). In addition, endothelial mitochondria enriched in NADPH oxidase 4 (NOX4) amplify oxidative signaling through ROS-induced ROS generation, particularly at mitochondria-associated membranes (MAMs), further exacerbating mitochondrial dysfunction and redox imbalance (48).

Endothelial mitochondrial content varies across vascular beds and with functional demands. Mitochondria volumes determined stereologically from electron micrographs suggested ECs within the blood-brain barrier possess higher mitochondrial density (8–11%) than ECs in quiescent capillary networks (27). Mitochondrial spatial distribution within ECs near the nucleus, mitochondria-associated endoplasmic reticulum (ER) membranes (MAMs), and cytoskeletal structures allows mitochondria to modulate endothelial responses to shear stress, hypoxia, and inflammatory stimuli (47). Moreover, mitochondrial perinuclear clustering and distribution within ECs influence mitochondrial signaling in the endothelium (44, 47). Overall, mitochondria in ECs primarily function as signaling organelles that regulate redox balance, calcium homeostasis, metabolic integration, and inflammatory responses.

Aging, sex, diet, and endothelial heterogeneity demonstrated to regulate mitochondrial function playing role in EC homeostasis. These factors regulate mitochondrial signaling in ECs through a coordinated control of redox balance, calcium handling, and metabolic signaling as described below:

#### 2.2.1 Role of aging in mitochondrial regulation of EC function

Mitochondrial morphology and dynamics are meticulously regulated throughout the ageing of cells. In senescent ECs (HUVECs), mitochondrial morphology showed elongated structure along with decreased membrane potential. The altered mitochondrial morphology was associated with suppression of fission factors (Drp1 and Fis1) and ROS induced fragmentation (41, 49). In addition, the proton leakage and increased calcium accumulation cause decreased membrane potential. Since the key role of EC mitochondria as an environmental sensor, dysregulation of mitochondrial quality control system affects ETC and decreases ATP production capacity. Furthermore, this exacerbates ROS production resulting in cell cycle arrest and promotes senescence-associated secretory phenotype (SASP) followed by proinflammatory environment for the ECs (50–52).

#### 2.2.2 Role of sex in mitochondrial regulation of EC function

Sex hormone based mitochondrial regulation plays one of the crucial roles of endothelial function. There is difference in mitochondrial Ca^2+^ handling capacity driven by sex hormones such as estradiol, testosterone. Female sex hormone estradiol exhibited higher Ca^2+^ storage capacity via increasing mitochondrial mass, membrane potential and reduced mitochondrial ROS compared to male sex hormone (testosterone). This estradiol driven superior mitochondrial Ca^2+^ handling facilitates the female endothelial nitric oxide synthase (eNOS) that leads to greater vasodilation (53). Also, Estrogen driven PGC1 regulates mitochondrial biogenesis that protect against ROS (54). However, in atherosclerotic model (Apoe^−/−^), female mice demonstrates opposite effects (55). Overall, studies suggest that sex hormones play functional role in mitochondria regulated EC homeostasis as evident in menopause.

#### 2.2.3 Role of diet in mitochondrial regulation of EC function

Western diet (WD) or high-fat diet (HFD) are rich in saturated fats and sugars. Since the last few decades, people are relying more on ultra-processed foods leading to increased fat deposition as adipose tissue in the body. Chronic intake of WD/HFD leads to obesity and insulin resistance, excess free fatty acids (FFAs) in circulation and systemic inflammation. As the immediate contact lining in circulation, ECs are exposed to those high blood sugar and FFAs (56, 57). When excess fatty acids and sugar inside the cell, there are more chances of ROS production due to incomplete metabolism. In addition, insufficient *β*-oxidation of excess fatty acids in mitochondria leads to oxidative stress. The cellular antioxidant defense fails to protect against this excess cellular oxidative stress. Consequently, this excess ROS impairs endothelial homeostasis by impairing eNOS bioavailability followed by membrane permeability. Mitochondria undergo alteration in their morphology and leads to the activation of apoptosis (58).

#### 2.2.4 Role of EC heterogeneity in mitochondrial regulation of EC function

ECs exhibit pronounced heterogeneity across vascular beds (e.g., brain, heart, lung), characterized by distinct transcriptional and functional signatures (59). Commonly used EC models, including human umbilical vein ECs (HUVECs), human dermal microvascular ECs (hdmvECs), and human foreskin microvascular ECs (hfmvECs), and organ-specific human pancreatic microvascular ECs (hpmvECs) exhibit significant differences in biochemical composition, relevant protein expression, angiogenic potential, and responses to mechanical shear stress (60). Comprehensive comparative analyses of Fifty-two purified EC populations isolated from arteries (aorta, coronary artery, pulmonary artery, iliac artery, and umbilical artery), veins (umbilical vein and saphenous vein), and tissues (skin, lung, intestine, uterus myometrium, nasal polyps, bladder, and myocardium) have demonstrated the diversity of EC phenotypes. Notably in this study, small vessel (microvascular) ECs appear to play a dominant role in regulating lipid transport and metabolic processes (61). Despite this extensive heterogeneity, the extent to which mitochondrial structure, dynamics, and signaling are differentially regulated across EC subtypes remains poorly defined. Comparative studies specifically addressing mitochondrial function under physiological and pathological conditions in distinct EC populations are limited. This represents a critical knowledge gap, as endothelial mitochondrial heterogeneity is likely a key determinant of vascular bed-specific susceptibility to dysfunction and atherosclerosis.

### 2.3 Mitochondrial involvement in regulating ROS and redox signaling

mtROS in ECs primarily originate from the ETC during oxidative phosphorylation, specifically at Complexes I and III. Superoxide (O_2_^•−^) is the proximal mtROS produced within the mammalian mitochondrial matrix. *In vitro* studies in isolated mitochondria showed that approximately 1–2% of the oxygen entering the ETC produces superoxide (O_2_^•−^), while the percentage attributable to ROS production in ECs *in vivo* remains unknown (62). In addition to ETC, mtROS is produced within the mitochondrial subcompartments by different sources. Within the mitochondrial matrix by metabolic enzymes such as aconitase (63), pyruvate dehydrogenase complex (PDH) (64), and *α*-ketoglutarate dehydrogenase complex (*α*-KGDH) (65); intermembrane space (IMS) by growth factor adapter protein p66Shc (66); the inner mitochondrial membrane (IMM) by nicotinamide adenine dinucleotide phosphate (NADPH) oxidase 4 (NOX4) (67) and mitochondrial ATP-sensitive potassium channel (66); and in the outer mitochondrial membrane (OMM) by monoamine oxidase (MAO) (68). mtROS generated from these different sources within the mitochondria under pathophysiological conditions contribute to EC senescence, migration, angiogenesis, and adaptive responses to hypoxia, inflammation, and oxidative stress.

Mitochondrial redox signaling in ECs functions as a double-edged sword, where low levels of ROS act as essential signaling messengers for vascular health, while excessive production drives vascular pathology. Under physiological conditions, ECs tightly regulate mtROS at a narrow physiological range, functioning as signaling molecules to regulate endothelial proliferation, migration, and adaptive responses to shear stress (69). However, pathological stimuli such as disturbed blood flow, hyperlipidemia, hyperglycemia, and chronic inflammation drive excessive mtROS generation and overwhelming antioxidant defenses. Excessive mtROS leads to direct cellular oxidative damage, activation of pathologic cell-signaling pathways, and, eventually, inactivation of protective mechanisms. Elevated mtROS also reduces NO bioavailability and inactivates NO by producing peroxynitrite, promoting eNOS uncoupling, and activating further ROS production. Excessive mtROS activates NF*κ*B and protein kinase C (PKC) signaling as a secondary mechanism, activating an array of proinflammatory and prothrombotic stimuli, altering the endothelial phenotype (70).

### 2.4 Mitochondrial involvement in regulating calcium signaling

Endothelial mitochondria play a central role in intracellular calcium (Ca^2^⁺) signaling by acting as dynamic buffers and relay stations. Studies using aortic ECs suggested that approximately 25% of intracellular Ca^2^⁺ is sequestered within mitochondria (43). Mitochondria within the ECs are localized near ER Ca^2^⁺ release sites and plasma membrane Ca^2^⁺ entry channels, which enables the formation of localized Ca^2^⁺ microdomains regulating the spatial and temporal Ca^2^⁺ signaling. For instance, stimulation of inositol 1,4,5-trisphosphate-generating agonists, endothelial mitochondria rapidly take up and release Ca^2^⁺, generating subplasmalemmal regions of reduced Ca^2^⁺ concentration. These microdomains sustain capacitative (store-operated) Ca^2^⁺ entry and facilitate Ca^2^⁺ transfer from the plasma membrane to the ER, thereby supporting prolonged signaling responses (71, 72). ECs stimulation generates a continuous Ca^2+^ flux through mitochondria, generating subplasmalemmal microdomains of low Ca^2+^, facilitating capacitative Ca^2+^ entry, and transferring Ca^2+^ from the plasma membrane to the ER (71, 72). NO further modulates mitochondrial Ca^2^⁺ handling in endothelial cells. Studies in cultured pulmonary artery endothelial cells indicate that NO derived from either plasma membrane-associated eNOS or mitochondria-localized NOS (mtNOS) can regulate mitochondrial Ca^2^⁺ uptake and efflux through negative feedback mechanisms (43). However, the effects of NO on mitochondrial Ca^2^⁺ homeostasis appear context-dependent and vary with cell type, experimental conditions, and NO concentration.

**Figure 2:**
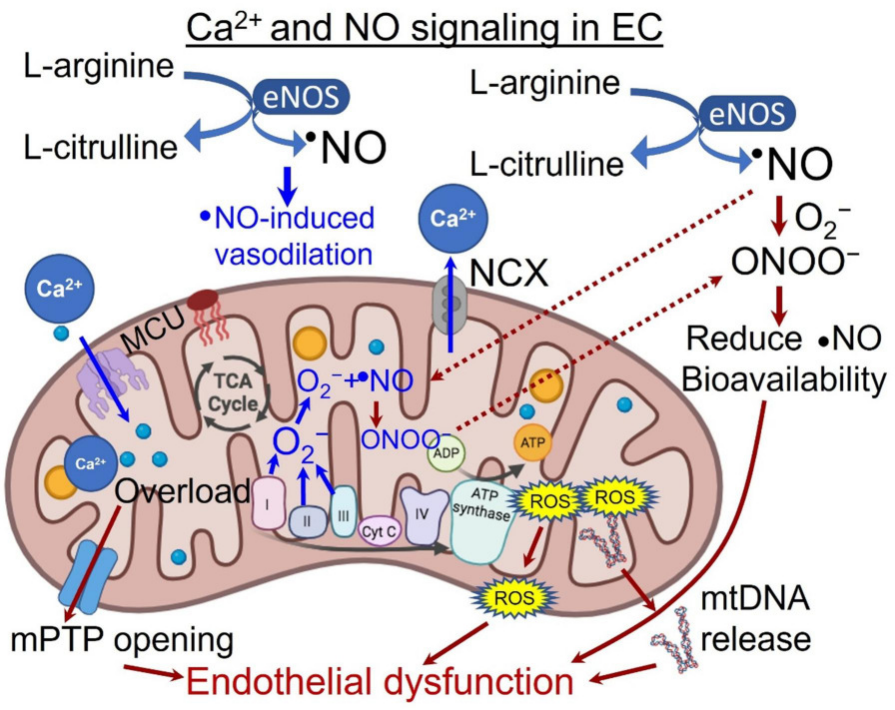
Ca²⁺ and NO signaling in endothelial mitochondria. Endothelial nitric oxide synthase (eNOS) catalyzes the conversion of L-arginine to NO and L-citrulline. Under physiological conditions (left), NO promotes vasodilation and supports vascular homeostasis. Mitochondrial Ca²⁺ uptake via the mitochondrial calcium uniporter (MCU) regulates tricarboxylic acid (TCA) cycle activity and oxidative phosphorylation, while Ca²⁺ extrusion through the Na⁺/Ca²⁺ exchanger (NCX) maintains calcium balance. Controlled mitochondrial Ca²⁺ flux sustains ATP production and limits reactive oxygen species (ROS) generation. Under pathological conditions (right), excessive mitochondrial Ca²⁺ accumulation leads to Ca²⁺ overload, enhanced electron transport chain (ETC) derived superoxide (O₂•⁻) production, and increased ROS formation. Superoxide reacts with NO to form peroxynitrite (ONOO⁻), reducing NO bioavailability and impairing vasodilation. Elevated ROS promotes mitochondrial permeability transition pore (mPTP) opening, mitochondrial DNA (mtDNA) release, and further oxidative damage. This feed-forward cycle of mitochondrial Ca²⁺ dysregulation, ROS amplification, NO depletion, and mtDNA release culminates in endothelial dysfunction.

Calcium enters to the mitochondria through mitochondrial calcium uniporter (MCU) complex comprising the pore-forming subunit MCU, regulatory subunits MCUR1, mitochondrial calcium uptake 1 (MICU1), MICU2, MICU3, MCUb, and EMRE. Mitochondrial calcium uniporter regulator 1 (MCUR1) is an essential inner mitochondrial membrane protein acting as a scaffold factor for the mitochondrial Ca^2+^ uniporter (MCU) complex. Deletion of MCU and MCUR1 in ECs impaired mitochondrial bioenergetics, cell proliferation, and migration (73). Similarly, MICU1 also acts as a critical gatekeeper in EC mitochondria, preventing Ca^2+^ overload, reducing oxidative stress, and maintaining vascular integrity (74, 75). In addition, oxidative stress (by H_2_O_2_) increases endothelial mitochondrial Ca^2+^ concentration [(Ca^2+^)m] by increasing transfer of cytosolic Ca^2+^, and inhibiting the mitochondrial Ca^2+^/Na^+^ exchanger (73). Disruption of mitochondrial Ca^2^⁺ handling promotes the key features of endothelial dysfunction and atherosclerosis, including mitochondrial depolarization, excessive ROS production, and endothelial activation (Figure 2).

## 3 Endothelial mitochondria as regulators of endothelial dysfunction

**Figure 3:**
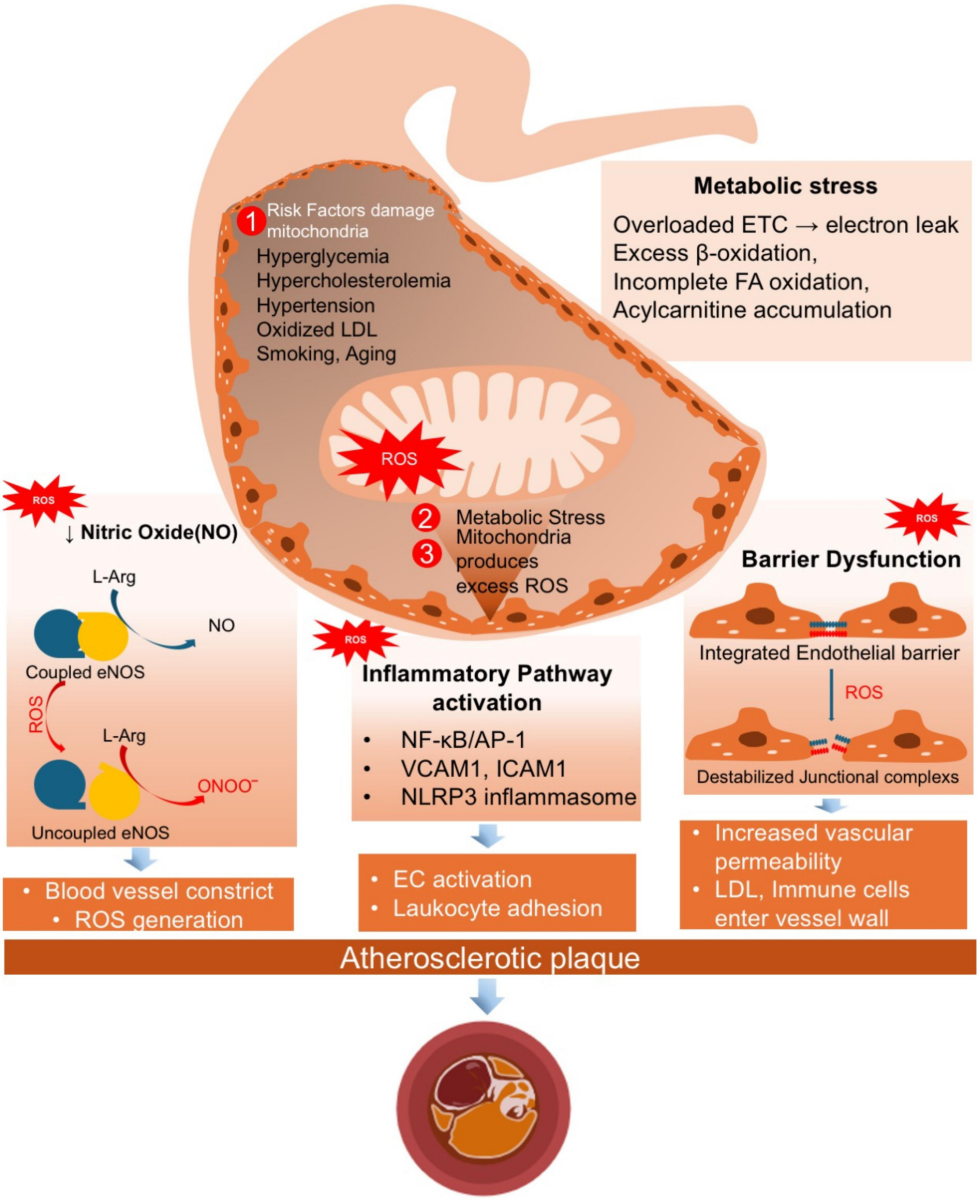
Endothelial mitochondrial dysfunction as a central driver of atherosclerosis. Cardiovascular risk factors (e.g., hyperglycemia, hypercholesterolemia, hypertension, oxidized LDL, aging, and smoking) impair endothelial mitochondrial function, leading to excess mitochondrial ROS production and metabolic stress. Mitochondrial ROS reduces nitric oxide (NO) bioavailability through eNOS uncoupling and peroxynitrite formation, disrupts endothelial barrier integrity by destabilizing junctional complexes, and activates redox-sensitive inflammatory pathways (NF-*κ*B/AP-1, NLRP3). These processes promote endothelial activation, leukocyte adhesion and transmigration, increased vascular permeability, and chronic inflammation, driving plaque formation, progression, and ultimately plaque rupture and thrombosis.

Endothelial mitochondria act as critical sensors and regulators of vascular health, where their dysfunction triggers endothelial dysfunction and subsequently initiates a series of events promoting atherosclerosis progression (76). Endothelial dysfunction is characterized by increased permeability, monocyte-leukocyte adhesion, and cytokine and chemokine production (77, 78). The underlying causes include imbalances in NO synthesis and bioavailability, mitochondrial dysfunction, increased oxidative stress, and increased inflammation (Figure 3).

### 3.1 Mitochondrial control of nitric oxide bioavailability

NO is a central determinant of endothelial homeostasis, regulating vascular tone and suppressing inflammation, thrombosis, and smooth vascular muscle cell proliferation (79–81). During atherogenesis, NO bioavailability is reduced through multiple mechanisms that converge on mitochondrial dysfunction. Depletion of NO bioavailability is associated with alterations in vascular tone, enhanced lipid accumulation, increased expression of cell adhesion molecules and platelet aggregation, and increased VSMC proliferation and migration toward the vessel lumen (81). The mechanisms that are involved in NO reduction include eNOS uncoupling, increased oxidative stress, and inflammation. NO is usually synthesized by eNOS in the endothelium using L-arginine and the cofactor tetrahydrobiopterin (BH4) (78). However, during atherosclerosis progression, BH4 levels are depleted by excessive oxidative stress, leading to increased superoxide production by eNOS rather than NO. eNOS is also uncoupled by S-glutathionylation of cysteine residues on eNOS enzyme, favoring more superoxide generation (82). Evidence indicates that eNOS inactivation is mediated by increased caveolin-1 synthesis, driven by higher oxidized LDL (oxLDL) cholesterol levels. Other factors that contribute to the depletion of NO levels include deficiency of eNOS substrate L-arginine, and endogenous inhibitor of eNOS asymmetric dimethylarginine (ADMA).

Mitochondrial signaling in HUVECs is tightly regulated by NO, which modulates the activity of hypoxia-inducible factor-1 (HIF-1*α*) and AMP-activated protein kinase (AMPK) independently of mitochondrial ATP synthesis. Experimental reduction of NO availability, either by pharmacological inhibition of NO production or by eNOS silencing, elevates intracellular oxygen (O_2_) levels, stabilizes HIF-1*α*, and enhances mtROS production, leading to AMPK activation and maintenance of a stress-resistant, non-angiogenic endothelial phenotype (83). Excessive generation of ROS reacts with NO to form another reactive molecule, peroxynitrite (ONOO-), leading to eNOS uncoupling and reduced NO bioavailability (84). The eNOS phosphorylation at serine 1,176 is crucial for its enzymatic activity. However, disruption of this phosphorylation can contribute to eNOS uncoupling, thereby triggering atherosclerotic plaque formation (85). These mitochondrial-dependent processes link metabolic stress directly to impaired NO signaling and endothelial dysfunction. The cardiovascular risk factors associated with impaired NO are high cholesterol, high blood pressure, diabetes, aging, smoking, and obesity. This dysfunction may lead to critical CVDs like hypertension, atherosclerosis, inflammation, and the formation of blood clots, increasing the risk for heart attack and stroke (86, 87).

### 3.2 Mitochondrial oxidative stress as a primary redox driver

An imbalance between ROS generation and antioxidant defenses may lead to excessive oxidative stress, a hallmark of atherosclerosis. In ECs, ROS are generated as byproducts of mitochondrial oxidative phosphorylation and function as second messengers in many physiological cellular processes. Atherogenic stimuli like hyperglycemia, hypercholesterolemia, hypoxia, and inflammation impair ETC function, particularly at complexes I and III, leading to excessive mitochondrial ROS production (88). ER stress can also contribute to mitochondrial dysfunction and ROS generation (89). Mitochondrial ROS can overwhelm the mitochondrial antioxidant defenses and promote ROS-induced ROS generation. In addition, during atherosclerosis progression, internalization of oxLDL via lectin-like oxLDL receptor 1 (LOX-1) in ECs triggers the receptor's activation, which then mediates NADPH oxidase activation and induces ROS accumulation (90, 91). Excessive superoxide in ECs may react with NO to generate reactive nitrogen species (RNS), such as ONOO-. Overproduction of both ROS and RNS is detrimental to EC function by inducing vascular permeability, activating inflammatory pathways, and disrupting vascular relaxation, ultimately leading to atherosclerosis (92, 93). Excess mtROS damages mitochondrial proteins, lipids, and mtDNA, disrupting bioenergetics and impairing mitochondrial turnover. This self-perpetuating cycle sustains endothelial oxidative stress and accelerates atherogenesis.

### 3.3 Mitochondria in the endothelial barrier integrity

Disruption of endothelial barrier integrity is an early and permissive event in endothelial dysfunction and atherosclerosis. Atherogenic stimuli increase vascular permeability, facilitating the entry of oxLDL, lipoproteins, and immune cells into the arterial wall and promoting inflammatory signaling and plaque formation (94). Barrier disruption is driven in part by phosphorylation and internalization of VE-cadherin and cytoskeletal remodeling via PKC/Src/RhoA signaling, leading to loss of tight and adherens junction integrity (95). Hemodynamic forces regulate endothelial barrier function. Unidirectional shear stress activates protective endothelial programs, whereas low or disturbed flow induces pro-atherogenic signaling pathways, including NF-*κ*B, AP-1, HIF-1*α*, and YAP/TAZ (96). Low shear stress in ECs impairs autophagy activity through reduced GABARAPL2 expression, destabilizes VE-cadherin-based junctions, and compromises barrier integrity via suppression of S1PR1 signaling (97). Inflammatory mediators and environmental stressors further disrupt tight junctions through MAPK/ERK and RhoA/ROCK pathways, leading to endothelial hyperpermeability and accelerating plaque progression (95, 98, 99).

### 3.4 Mitochondria in endothelial activation and inflammation

Mitochondria in ECs act as critical hubs for inflammatory and innate immune signaling by activating the NLRP3 inflammasome and initiating inflammatory responses through multiple mechanisms, including mtDNA leakage, mtROS production, ATP signaling, and interactions with lysosomes (100). Under physiological conditions, mtDNA is confined within mitochondria; however, mitochondrial dysfunction caused by cellular stress or injury leads to the release of mtDNA into the cytoplasm, where it functions as a damage-associated molecular pattern (DAMP) (101). Cytosolic mtDNA activates the DNA sensor cyclic GMP-AMP synthase (cGAS), leading to activation of the stimulator of interferon genes (STING) signaling pathway (102). Upon activation, STING translocates from the ER to the Golgi apparatus, recruits TANK-binding kinase 1 (TBK1) and activates nuclear factor-*κ*B (NF-*κ*B) and interferon regulatory factor 3 (IRF3). IRF3 induces the transcription of type I interferons, while NF-*κ*B promotes the expression of pro-inflammatory cytokines such as TNF*α* and IL-6 (100). Studies in human aortic endothelial cells showed the activation of the mtDNA-cGAS-STING-IRF3 signaling axis is critically involved in metabolic stress-induced mitochondrial damage and endothelial inflammation (103). In addition, activation of the cGAS-STING pathway during viral infections in epithelial and endothelial cells promotes macrophage-driven immune pathology. It facilitates T-lymphocyte transendothelial migration in TNF*α*-induced peritonitis in both mouse and human ECs (104, 105).

Mitochondrial dysfunction, mtROS accumulation, and mtDNA release also promote activation of the NLRP3 inflammasome in macrophages (106). Leaked mtDNA can directly interact with the NLRP3 inflammasome or indirectly activate NLRP3 via the cGAS-STING pathway. Additional mitochondrial components involved in NLRP3 inflammasome activation include mitochondrial antiviral-signaling protein (MAVS) and the mitochondrial-specific phospholipid cardiolipin. MAVS promotes NLRP3 inflammasome activation through direct interaction with NLRP3 and by recruiting TRAF3 to ASC, thereby facilitating ASC oligomerization and inflammasome assembly (107, 108). Similarly, externalized cardiolipin binds directly to the leucine-rich repeat (LRR) domain of NLRP3, and disruption of cardiolipin biosynthesis impairs NLRP3 inflammasome activation (109, 110). Collectively, these mitochondrial signaling pathways initiate inflammatory responses, promote endothelial dysfunction, and modulate cellular responses to metabolic stress and infection.

ECs serve as active regulators of immune cell recruitment in response to metabolic and oxidative stress, thereby initiating atherosclerosis pathogenesis. An array of atherogenic stimuli, including oxLDL, ROS, proinflammatory cytokines (e.g., TNF*α*, IFN*γ*, IL-1β), and chemokines (e.g., MCP-1), activate endothelial inflammatory signaling (e.g., NF-*κ*B and AP-1). These atherogenic stimuli lead to increased expression of adhesion molecules (VCAM1, ICAM1, and E-selectin), promoting leukocyte rolling, adhesion, and transmigration into the vessel wall. Endothelial inflammatory responses are further amplified by activation of TLR4, MAPK, and NLRP3 inflammasome pathways, thereby initiating immune cell recruitment and plaque development (111–113). In addition, Wnt signaling has also emerged as an important regulator of atherosclerosis progression. The Wnt signaling sustains plaque growth and instability through the modulation of endothelial dysfunction, monocyte infiltration, smooth muscle cell migration, and macrophage-driven inflammation (114).

## 4 Defects in endothelial mitochondrial quality control machinery in atherosclerosis

Like other cell types, endothelial mitochondria are highly dynamic organelles forming interconnected networks undergoing continuous fission, fusion, transport, and selective degradation of damaged mitochondria. Cells maintain mitochondrial homeostasis by an elaborate and sophisticated mechanism to remove the damaged or unwanted mitochondria via a process termed mitophagy. These quality control processes are essential for maintaining mitochondrial function and endothelial homeostasis. Accumulation of dysfunctional mitochondria within ECs can contribute to the signaling cascade underlying EC dysfunction, which may ultimately initiate and promote atherosclerosis. EC dysfunctions are characterized by impaired energy metabolism, increased oxidative stress, and increased activation of inflammation signaling pathways. Recent findings in the literature suggest mitochondrial dysfunction is a major contributor to the development and progression of atherosclerotic pathology. Therefore, understanding the role of mitochondrial dysfunction in ECs and the underlying mechanisms is crucial for identifying potential therapeutic targets to prevent vascular diseases.

### 4.1 Role of excessive mitochondrial-derived reactive oxygen species (mtROS)

Endothelial mitochondria act as both sources and targets of oxidative stress in response to pathological stressors. Excessive mtROS damage ETC components and oxidatively modify mitochondrial proteins, lipids, and DNA, impairing their function. Important enzymes that are localized in mitochondria, including pyruvate dehydrogenase, respiratory complex (I-III), and ketoglutarate dehydrogenase, are sensitive to oxidative stress and become inhibited. Oxidative modifications of endogenous mitochondrial antioxidants inhibit their enzymatic functions and lead to a decrease in ROS scavenging (115). Overproduction of ROS activates proinflammatory and prothrombotic pathways in ECs. Overabundance of ROS reduces the bioavailability of NO by oxidizing it to another potent oxidant, ONOO-, and uncoupling eNOS. Excessive ONOO^−^ can induce nitrosative stress to the cells, further aggravating EC dysfunction. Oxidative stress in cells contributes to the activation of inflammatory pathways in ECs, which are among the initial steps in atherosclerosis development, including the NF*κ*B and NLRP3 inflammasome pathways. mtDNA is more vulnerable to ROS-induced damage than genomic DNA since it lacks protective histone and repair capabilities (116). ROS can also induce additional mtDNA mutations, promoting transcriptional defects in mitochondrial ETC complex subunits, thereby decreasing energy production and increasing ROS generation. Damaged mtDNA also triggers the opening of mitochondrial permeability transition pores (MPTP), causing outer membrane permeability and the release of apoptogenic proteins into the cytosol (117). Mitochondrial ROS also damages endothelial barrier integrity, leading to hyperpermeability. This leads to cytochrome C release from the mitochondria to activate caspase-3, which then disrupts the veCadherin-*β*-catenin complex in the adherens junctions by cleaving *β*-catenin (118, 119). Collectively, mtROS-induced damage to mitochondrial proteins and mtDNA further impairs ETC function, reinforcing ROS generation and endothelial dysfunction.

### 4.2 Role of dysregulated mitochondrial dynamics

Dysregulated mitochondrial dynamics resulting from the imbalance of mitochondrial fission and fusion are a central driver of endothelial dysfunction, vascular inflammation, and atherosclerosis. Vascular risk factors that have been found to trigger impaired mitochondrial dynamics include hyperglycemia, excessive ROS, oxLDL, and LPS (5). Hyperglycemia in diabetic patients is associated with endothelial dysfunction and altered mitochondrial morphology, resulting from increased mitochondrial fission. Researchers have shown that excessive cytosolic and mitochondrial ROS can affect fission regulatory protein DRP1 phosphorylation and upstream regulators in ECs, including ERK, JNK, and PKC, thereby increasing mitochondrial fragmentation and dysfunction (120–123). High glucose levels can also impair mitochondrial oxidative phosphorylation, thereby reducing mitochondrial fusion in ECs (124). This increased mitochondrial fission can, in turn, lead to greater mitochondrial ROS production in ECs during diabetes, ischemia/reperfusion injury, or disturbed flow in atheroprone regions (125). OxLDL induces DRP-1-mediated mitochondrial fission, which then increases endothelial apoptosis, possibly by a direct interaction between DRP1 and microRNAs (e.g., miR-199b-5p) (126). However, the mechanism by which oxLDL regulates mitochondrial fission remains unclear. Additionally, oxLDL promotes mitochondrial senescence in ECs by suppressing DRP1 levels (127). LPS is an inflammatory trigger for many vascular diseases, such as atherosclerosis, and can induce mitochondrial fission by altering DRP1 phosphorylation and mitochondrial ROS generation (128, 129).

Endothelial cells from diabetic patients and high-glucose-treated human aortic endothelial cells exhibit increased FIS1 expression, mitochondrial fragmentation, impaired eNOS signaling, and elevated ROS; genetic or pharmacological inhibition of DRP1 or FIS1 reverses these defects (130). Excessive mitochondrial fission is also linked to NF-*κ*B activation, leukocyte adhesion, and endothelial inflammation, effects that are attenuated by DRP1 inhibition *in vitro* and *in vivo* (129, 131). Conversely, impaired mitochondrial fusion exacerbates disease, as loss of OPA1 in ECs accelerates atherosclerosis in LDLr⁻^/^⁻ mice (132). Beyond ECs, mitochondrial dynamics influence plaque stability. The circular RNA circHIPK3 is enriched in vulnerable plaques and interacts with DRP1 to enhance mitochondrial fission, increasing ROS, impairing mitochondrial function, and triggering necroptosis in vascular smooth muscle cells (133). *In vivo* silencing of circHIPK3 by delivery of AAV-shRNA targeting circHIPK3 reduces plaque vulnerability and slows atherosclerotic progression (133).

### 4.3 Role of defective mitophagy

Mitophagy eliminates damaged mitochondria, which may lead to EC dysfunction. The ubiquitin-dependent pathway of mitophagy is regulated by the PINK1 (phosphatase and tensin homolog-induced putative kinase 1) and parkin (E3 ubiquitin ligase) signaling pathway. PINK1 accumulates on damaged mitochondria and recruits Parkin to the damaged mitochondria, which attaches ubiquitin tags to mitochondrial proteins (such as Mfn2, VDAC1, and Miro), and subsequently is recognized by autophagy receptors [e.g., microtubule-associated protein 1 light chain 3 (LC3), nuclear dot protein 52 (NDP52), and Optineurin (OPTN)], promoting their degradation (134, 135). PINK1/ Parkin pathway-mediated mitophagy protects ECs from metabolic stress-induced mitochondrial damage and cell death (136). Ubiquitin-independent pathways of mitophagy are mediated by proteins such as NIP3-like protein X (NIX), BCL2-interacting protein 3 (BNIP3), prohibitin 2 (PHB2), and FUN14 domain-containing 1 (FUNDC1), which bind directly to damaged mitochondria and promote their entry into the autophagosome (137–139). EC-specific FUNDC1 deficiency impaired basal mitophagy flux, resulting in dysfunctional mitochondria accumulation, metabolic reprogramming toward aerobic glycolysis, pseudohypoxia, and senescence, resulting in pulmonary hypertension in mice (139). Dysregulation of mitochondrial dynamics and mitophagy leads to accumulation of dysfunctional mitochondria, sustained oxidative stress, and pro-inflammatory endothelial phenotypes (140).

Emerging evidence links impaired endothelial mitochondrial quality control to vascular aging, plaque development, and progression of atherosclerotic cardiovascular disease. Risk factors for atherosclerosis, including a high-fat, high-cholesterol diet, hyperglycemia, oxLDL, ROS, and hypoxia, can also trigger activation of mitophagy. However, if activated mitophagy is insufficient to eliminate damaged mitochondria and restore homeostasis, the damage persists, leading to worsening atherosclerosis. However, inadequate or dysregulated mitophagy fails to restore mitochondrial homeostasis, thereby exacerbating endothelial dysfunction and disease progression. Hyperglycemia and excessive ROS decrease mitophagy in ECs in diabetic rats by reducing mitophagy regulatory proteins, PINK1, Parkin, and LC3B, resulting in the accumulation of defective mitochondria and EC dysfunction (141, 142). High fat, on the other hand, leads to excessive mitophagy mediated by Parkin phosphorylation and EC apoptosis (143). Hypoxia activates mitophagy through the HIF1*α*-BNIP3-FUNDC1 pathway in HUVECs and plays a protective role in hypoxia-induced EC damage by maintaining mitochondrial quality (144, 145).

### 4.4 Role of mitochondrial DNA (mtDNA) damage

mtDNA is highly susceptible to oxidative injury due to the absence of histone protection and limited DNA repair capacity. Excessive mitochondrial ROS promotes mtDNA damage and mutations, disrupting mitochondrial gene expression, impairing electron transport chain function, and reducing ATP production (146, 147). When damaged mtDNA is released into the cytosol or circulation, it functions as a mitochondrial DMAP (mtDAMP), activating innate immune signaling and driving endothelial inflammation and atherosclerotic plaque progression (148). Experimental models support a strong link between mtDNA injury and cardiovascular disease. In high-fat diet-fed ApoE^−/−^ mice, mtDNA damage and reduced mtDNA copy number correlate with decreased ETC complex subunits (I, III, IV, and V) and mitochondrial dysfunction, which led to abnormal proliferation, migration, and inflammation of vSMCs in the atherosclerotic plaque (149). mtDNA damage leads to the opening of the MPTP, causing outer-membrane rupture. When damaged mtDNA is leaked out from mitochondria into the cytosol and eventually into the bloodstream, it acts as DAMPs and induces inflammation, promoting atherosclerotic plaque formation (148). mtDNA damage also alters mitochondrial membrane potential, reduces ATP generation, and triggers mitophagy by upregulating PINK1 kinase in atherosclerotic mice (149).

### 4.5 Role of mitochondria-derived signals

Bioactive signals released from dysfunctional mitochondria promote vascular inflammation and atherosclerosis, including mtDAMPs, metabolites, and peptides. mtDAMPs, such as mtDNA, cytochrome c, HSP60, TFAM, cardiolipin, succinate, and N-formyl peptides, activate proinflammatory signaling in vascular cells and accelerate endothelial dysfunction (150–152). ECs are particularly sensitive to these signals, as mtDAMPs can originate both intracellularly and from the circulation. In addition to mtDAMPs, mitochondria-derived peptides modulate endothelial responses. Circulating factor 6 (CF6) is released from mitochondria and endothelial membranes and promotes inflammatory activation, whereas peptides such as humanin and prohibitin-1 exert protective effects by limiting oxLDL-induced oxidative stress and endothelial apoptosis (153, 154).

## 5 Targeting endothelial mitochondrial homeostasis in atherosclerosis

**Figure 4:**
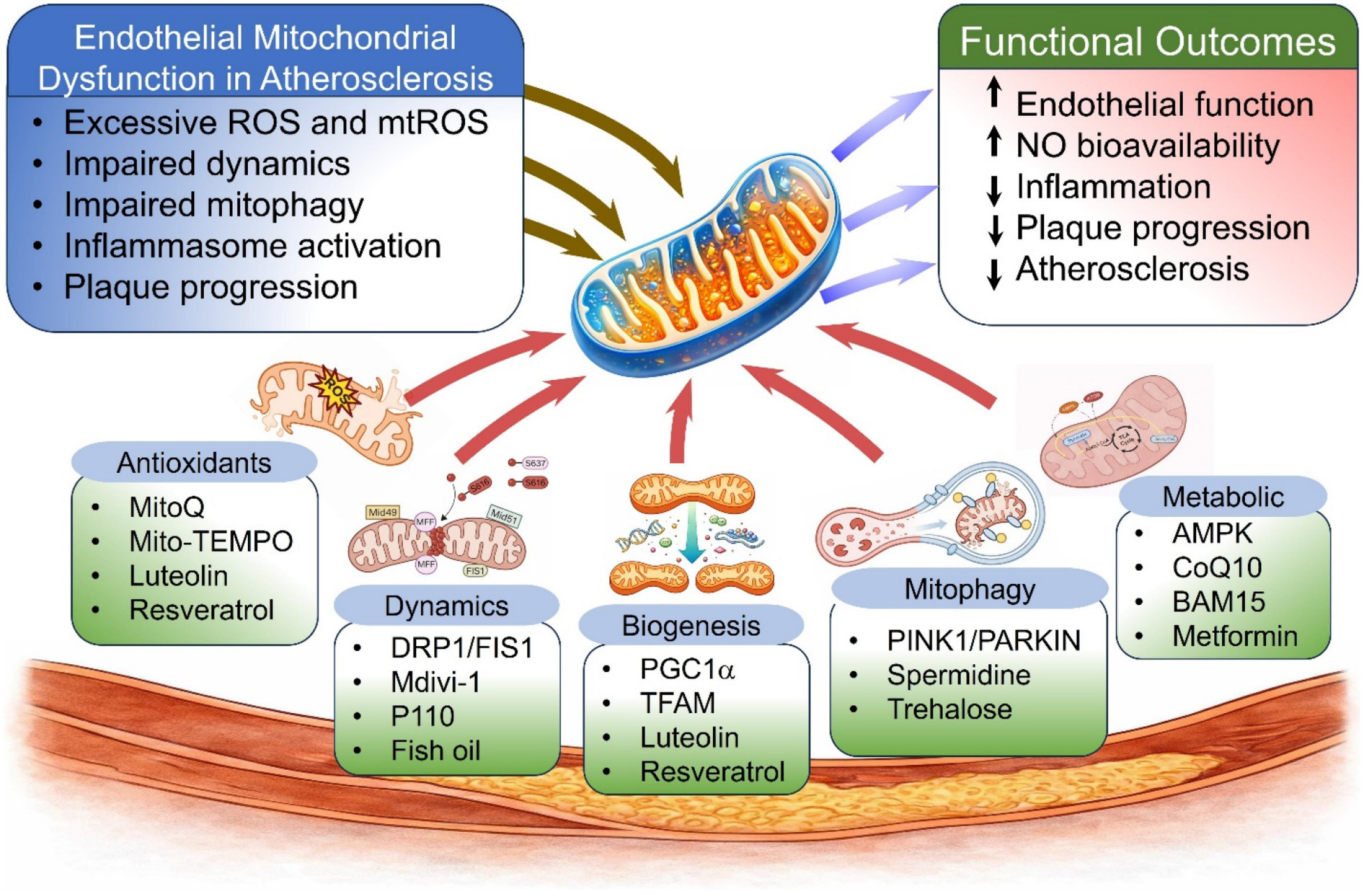
Therapeutic targeting of endothelial mitochondrial homeostasis in atherosclerosis. Endothelial mitochondrial dysfunction in atherosclerosis: excess mitochondrial reactive oxygen species (mtROS), Ca²⁺ overload, impaired mitophagy, disrupted mitochondrial dynamics (DRP1/Fis1-dependent fission), mitochondrial DNA (mtDNA) damage, inflammasome activation, endothelial dysfunction, and plaque progression. Therapeutic mitochondrial reprogramming: redox modulation (MitoQ, MitoTEMPO, luteolin, resveratrol); mitochondrial dynamics (DRP1/Fis1 inhibition by Mdivi-1, P110; fusion restoration); mitophagy and quality control (PINK1/Parkin-receptor-mediated pathways, spermidine, trehalose); mitochondrial biogenesis and transcriptional control (PGC1*α*, NRF1, TFAM, ERR*α*); metabolic and energy-sensing pathways (AMPK activation, metformin, statins, polyphenols, CoQ10/ubiquinol, BAM15); mitochondrial inflammatory signaling (mtROS/NLRP3/caspase-1/IL-1β axis). Functional outcomes: increased NO bioavailability, reduced vascular inflammation, improved endothelial function, plaque stabilization, attenuation of atherosclerosis.

Mitochondrial dysfunction is very closely associated with oxidative damage, altered calcium homeostasis, and disrupted ATP synthesis. To restore mitochondrial function, these features must be corrected by targeting both the inner and outer mitochondrial membranes. To date, a variety of methods have been used to alleviate mitochondrial dysfunction, including behavioral approaches (e.g., exercise, lifestyle, diet, and vitamin supplementation) and therapies such as antioxidants, gene therapy targeting mtDNA, and anti-inflammatory agents. Recently, targeting mitochondrial proteins in atherosclerosis treatment has emerged as a promising approach, given mitochondrial involvement in cellular metabolism, inflammation, and ROS production, which play critical roles in atherogenesis (Figure 4).

### 5.1 Targeting mitochondrial antioxidants and redox modulation

Excessive mtROS production is a central driver of endothelial dysfunction in atherosclerosis. Consequently, antioxidant therapies targeting mitochondria have gained considerable attention. Mitochondria-targeted antioxidants (MitoQ and MitoTEMPO) selectively scavenge mitochondrial superoxide and restore redox balance in experimental animal models. In preclinical studies, **MitoQ** demonstrated EC-protective effects in several animal models of vascular dysfunction, including spontaneously hypertensive stroke-prone rats (126), doxorubicin-treated mice (127), and age-related endothelial dysfunction (155, 156). Similarly, MitoQ treatment has been shown to improve endothelial dysfunction in patients with peripheral artery disease (157), chronic obstructive pulmonary disease, and aging-associated arterial stiffness in older adults (158, 159). Multiple ongoing clinical trials are investigating whether MitoQ can improve vascular function across a range of pathological conditions, including studies in patients with peripheral artery disease (NCT03506633), chronic kidney disease (NCT02364648), and heart failure with preserved ejection fraction (NCT03960073). **Mito-TEMPO** (triphenylphosphonium chloride) is a superoxide dismutase (SOD) mimic that functions as a mitochondria-targeted antioxidant with potent superoxide and alkyl radical-scavenging activity (160). Treatment with mitoTEMPO shown to improve endothelium-dependent dilation in arterioles isolated from adipose tissue biopsies in patients with type 2 diabetes (161) and microvascular function in patients with chronic kidney disease (162). **Luteolin**, a flavonoid antioxidant, suppresses H_2_O_2_-mediated oxidative stress and inhibits mtROS-driven p38/MAPK/NF-*κ*B inflammatory signaling. In addition to reducing oxidative stress, luteolin modulates intracellular calcium levels, mitochondrial membrane potential, cytochrome *c* release, and apoptosis, ultimately improving endothelial function (163, 164). **Resveratrol** similarly exerts atheroprotective effects by promoting mitochondrial fusion, reducing mtROS production, and regulating the function of mitochondrial membrane proteins (165). Other antioxidant compounds, including ilexgenin A, salidroside, and corylin, have demonstrated protective effects against atherosclerosis by attenuating mitochondrial oxidative stress and preserving EC function (166).

### 5.2 Targeting mitochondrial dynamics (fission, fusion, and structural integrity)

Disruption of mitochondrial dynamics is a key contributor to endothelial dysfunction and to the progression of atherosclerotic disease. Excessive mitochondrial fission, largely driven by aberrant activation of DRP1/Fis1, promotes mitochondrial ROS production, inflammasome activation, and endothelial inflammation (167, 168). ECs isolated from patients with diabetes exhibit increased mitochondrial fragmentation, accompanied by elevated expression of Fis1, compared with healthy controls (130). Consistently, exposure of human aortic ECs to high glucose disrupts mitochondrial network integrity, increases DRP1 and Fis1 expression, elevates mitochondrial ROS levels, and markedly impairs agonist-stimulated eNOS activation (130). Loss of protein disulfide isomerase A1 (PDIA1) in ECs exacerbates mitochondrial fragmentation and mtROS accumulation by enhanced sulfenylation of DRP1 at Cys644, leading to increased DRP1 activity, endothelial senescence, and impaired endothelium-dependent vasorelaxation and angiogenesis (169). In contrast, mitochondrial fusion protein mitofusin-2 (Mfn2) expression is progressively reduced in the arteries of ApoE⁻^/^⁻ mice during atherogenesis (170), and restoring Mfn2 levels attenuates plaque formation through modulation of Akt and ERK signaling pathways (170).

Pharmacological inhibition of DRP1-dependent mitochondrial fission has shown significant vascular benefits. The DRP1 inhibitor **Mdivi-1** reduces atherosclerotic burden by suppressing mitoROS-driven NLRP3 inflammasome activation and M1 macrophage polarization (171). Similarly, **P110**, a selective inhibitor of the DRP1-Fis1 interaction, limits excessive mitochondrial fragmentation, reduces mtROS production, and confers cytoprotection in ECs exposed to inflammatory stressors such as lipopolysaccharide (128, 172). Metabolic modulators also influence mitochondrial dynamics. Both **metformin** and **resveratrol** suppress DRP1-mediated mitochondrial fission and inhibit ER stress-associated NLRP3 inflammasome activation in diabetic models (173). In streptozotocin-induced diabetic ApoE⁻^/^⁻ mice, metformin markedly reduces mitochondrial fragmentation and superoxide production, improves endothelium-dependent vasodilation, dampens vascular inflammation, and limits atherosclerotic lesion development (174). Consistently, treatment with mdivi-1 alleviates oxidative stress, improves endothelial function, suppresses inflammation, and attenuates atherosclerosis in diabetic mice (174). Dietary interventions, including **fish oil** supplementation, also modulate proteins regulating mitochondrial dynamics and protect against high-fat diet-induced endothelial dysfunction (175).

### 5.3 Targeting mitophagy and mitochondrial quality control

Efficient mitophagy is essential for the removal of damaged mitochondria and the preservation of endothelial homeostasis. Although excessive mitophagy may be detrimental, the controlled activation of mitochondrial quality-control pathways represents a promising therapeutic avenue. Fine-tuning mitophagy to eliminate damaged mitochondria without compromising mitochondrial mass is likely critical for maintaining vascular health. **Spermidine**, a dietary polyamine abundant in soy products and certain aged cheeses, has been shown to stimulate autophagy and mitophagy (176, 177). In mice, spermidine treatment enhanced endothelial function in aged mice, reduced atherogenesis by enhancing mitophagy, and attenuated mitochondrial dysfunction (178), and attenuated age-related increases in aortic stiffness (176). **Trehalose**, a dietary disaccharide found in mushrooms and honey, promotes autophagy and mitophagy (179). In aging and cardiometabolic disease models, trehalose-induced mitophagy is associated with improved endothelial function and reduced arterial stiffening (180–183). These vascular benefits are linked to enhanced mitochondrial quality control, including suppression of p66Shc signaling, lower oxidative stress, increased resistance to mitochondrial stress, and improvements in nitric oxide-dependent endothelial function (180–183).

Although excessive mitophagy can be detrimental by promoting mitochondrial depletion and bioenergetic failure, inhibition of mitophagy does not confer protection in atherosclerosis and generally exacerbates disease progression. However, emerging evidence indicates that the effects of modulating mitophagy may be context- and cell type-dependent. For example, coenzyme Q10 has been reported to inhibit mitophagy while attenuating nucleoside reverse transcriptase inhibitor (NRTI)-induced endothelial dysfunction in human aortic and umbilical vein endothelial cells (184). Conversely, in LPS/ATP-stimulated RAW264.7. macrophages, coenzyme Q10 enhances mitophagy and suppresses NLRP3 inflammasome activation under inflammatory stimulation (185). These findings highlight the complexity of mitophagy regulation across different vascular cell types and disease contexts, underscoring the need for precise, cell-specific therapeutic strategies targeting mitochondrial quality control in atherosclerosis.

### 5.4 Targeting mitochondrial biogenesis and transcriptional control

Enhancing mitochondrial biogenesis is another strategy to restore endothelial mitochondrial homeostasis. Transcriptional regulators such as PGC-1*α*, PGC-1*β*, NRF1, ERR*α*, and TFAM coordinate mitochondrial DNA replication, oxidative phosphorylation, and antioxidant defense. Genetic disruption of mitochondrial biogenesis pathways, including deletion of *TFAM* or *AIFM1*, results in severe mitochondrial dysfunction and cardiomyopathy (186, 187). Metabolic regulators such as NRF2F2 and HIF-1*α* also influence mitochondrial adaptation in cardiovascular disease. While NRF2F2 is upregulated in heart failure, HIF-1*α* activation promotes PPAR*γ* signaling, increasing glucose uptake, lipid accumulation, and apoptotic cardiomyocyte death (188). Modulating these transcriptional programs offers opportunities to improve mitochondrial resilience in atherosclerosis.

### 5.5 Targeting mitochondrial metabolism and energy-sensing pathways

Targeting cellular metabolic pathways that regulate mitochondrial function has shown benefit in cardiovascular disease. **AMPK** is a central energy sensor that coordinates mitochondrial biogenesis, fatty acid oxidation, and redox balance. Pharmacological agents such as statins, metformin, polyphenols, and PPAR*γ* agonists converge on AMPK activation, leading to reduced ROS production, suppression of inflammation, and improved endothelial function (189). AMPK activation also inhibits DRP1-mediated mitochondrial fission and suppresses ROS-dependent NLRP3 inflammasome activation, thereby protecting endothelial homeostasis (190). **Coenzyme Q10 (CoQ10)**, a key component of the mitochondrial ETC, activates AMPK by suppressing YAP signaling, reducing ROS generation, inducing OPA1 expression, and preserving mitochondrial integrity (191). Long-term coenzyme Q10 supplementation has been associated with enhanced endothelial function in multiple clinical and nonclinical settings (192). Beneficial effects have been documented in patients with ischemic heart disease, individuals with type 2 diabetes, and otherwise healthy subjects presenting with impaired endothelial function (193–196). More recent studies indicate that ubiquinol, the reduced and biologically active form of coenzyme Q10, improves endothelial function, as measured by microvascular perfusion, in patients with antiphospholipid syndrome. These vascular benefits are thought to arise, at least in part, from improved mitochondrial function and attenuation of oxidative stress (197). **BAM15** is a novel mitochondrial uncoupler that has shown preclinical promise in restoring mitochondrial function, improving glucose and lipid metabolism, and protecting ECs from hyperglycemia-induced damage (198). BAM15 reduces mitochondrial damage, lowers ROS production, inhibits apoptosis, and promotes FAO, collectively contributing to vascular protection in diabetes-associated atherosclerosis (199).

### 5.6 Targeting mitochondrial inflammation

The NLRP3 inflammasome is tightly linked to mitochondrial dysfunction during atherosclerosis. Triggers for inflammasome activation include FA-induced mitochondrial uncoupling, excessive mitochondrial fission, NADPH oxidase activation, and mtROS accumulation (200, 201). In preclinical studies, UCP1 deficiency exacerbates dietary obesity-induced endothelial dysfunction, vascular inflammation, and atherogenesis by augmenting mitochondrial membrane potential and mitochondrial superoxide, leading to hyperactivation of the NLRP3-inflammasome and caspase-1-mediated maturation of interleukin-1β (IL-1β) (202). In this study, treatment with BAM15 conferred protection against obesity-induced vascular inflammation and atherosclerosis by blocking the mtROS/NLRP3 inflammasome/caspase-1/IL-1β signaling axis (202). Statin treatment has been shown to suppress mtROS production, reduce NLRP3 inflammasome activation, and decrease IL-1β and caspase-1 levels, resulting in reduced myocardial injury and fibrosis *in vivo* (203). Therefore, targeting mitochondrial-driven inflammatory signaling represents a key strategy to limit endothelial inflammation and plaque progression.

### 5.7 Emerging strategies: nano-therapy and mitochondrial targeting

Recent advances in nanotechnology have enabled the development of mitochondria-targeted delivery systems. Mitochondria-targeted high-density lipoprotein mimetic nanoparticles have been engineered to deliver therapeutic cargo to the mitochondrial matrix and intermembrane space, reducing cholesterol and triglyceride accumulation in preclinical models (204). Despite promising results, achieving precise and efficient mitochondrial targeting *in vivo* remains challenging, underscoring the need for continued investigation into endothelial-specific mitochondrial biology.

Collectively, targeting mitochondrial antioxidants, dynamics, biogenesis, mitophagy, metabolic signaling, and inflammatory pathways offers a multifaceted approach to restoring endothelial mitochondrial homeostasis in atherosclerosis. Tables 1, 2 summarize key mitochondrial genes, pathways, and therapeutic strategies that have been explored to modulate mitochondrial function in cardiovascular disease.

**Table 1:** Mitochondria-targeted genes and pathways implicated in atherosclerosis.

Key genes/ Regulators	Pathway	Mechanistic role in atherosclerosis	Citations
Mitochondrial biogenesis and transcriptional control			
PGC1*α*, PGC1*β*, NRF1, ERR*α*	Mitochondrial biogenesis and antioxidant defense	Upregulation promotes mitochondrial biogenesis and antioxidant activity. Prevents atherosclerotic lesion formation and endothelial dysfunction.	(205, 206)
AIFM1, TFAM	Mitochondrial metabolism	Protects mitochondrial DNA and oxidative phosphorylation. Deletion leads to cardiomyopathy.	(186, 187)
NRF2F2, HIF1*α*	Mitochondrial energy regulation	Regulate metabolic adaptation, glucose uptake, lipid accumulation, and apoptosis in cardiomyocytes.	(188)
Pparα, Ppar*γ*	Lipid Metabolism And Ppar Signaling	Regulate Mitochondrial Genes And Lipid Oxidation, And The Dysfunction Of These Proteins.	(186, 187)
Metabolic Regulation and Energy-Sensing Pathways			
AMPK	Cellular energy sensing and metabolic regulation	Key energy sensor regulating mitochondrial biogenesis and ROS reduction.	(207, 208)
Mitochondrial dynamics and mitophagy regulatory pathways			
DRP1, MFN1, MFN2, OPA1, PINK1, Parkin	Mitochondrial dynamics (fission and fusion) and mitophagy	Excessive fission and/or impaired fusion facilitate endothelial and smooth muscle cell dysfunction. Overexpression of OPA1 rescues oxLDL-induced damage.	(208)
Reactive oxygen species (ROS) and redox-modulating pathways			
ETC complexes, MAO-A, SOD2	ROS and redox signaling	mtROS stimulates NF*κ*B activation, endothelial and macrophage inflammation, and vascular dysfunction.	(207, 208)
METTL4, MT-ATP6	mtDNA damage and epigenetics	METTL4 mediates 6 mA modification of mitochondrial DNA by suppressing transcription of mitochondrial ATP6. This affects ETC activities, increases mtROS generation, and promotes macrophage inflammation during atherosclerosis.	(208)
Mitochondria-linked inflammatory pathways			
NLRP3 inflammasome	Inflammasome activation and mtDNA release	MPTP opening and releasing mtDNA from damaged mitochondria activate NLRP3 inflammasome signaling and promote vascular inflammation.	(208, 209)
NFκB, MAPK, TLRs, MAVS, IRF3	Mitochondria-linked inflammatory pathways	DAMPs activate PRP and inflammatory signaling cascades in EC. Anti-inflammatory agents and antioxidants can inhibit this.	(210)

**Table 2:** Mitochondria-targeted therapies to treat atherosclerosis.

Drug/Strategy	Target/Mechanism	Mechanistic effects on atherosclerosis or vascular function	Citations
Resveratrol/ Astragaloside IV	Activate PGC1α, promote fusion, and reduce ROS	Improve mitochondrial function and prevent vascular apoptosis	(166, 211)
Cyclosporine A	Cyclophilin D inhibitor to prevent MPTP opening	Decreases mitochondrial-induced apoptosis	(212)
MitoQ, CoQ10, MitoVit-E	Mitochondria-targeted antioxidants	Scavenge mitochondrial ROS, restore redox balance, and improve endothelial function by decreasing oxidative stress	(205)
DRP1 inhibitors, OPA1 activators	Balancing mitochondrial fission-fusion dynamics	Improve mitochondrial functions in endothelial and vascular smooth muscle cells	(166)
Pemetrexed	METTL4 inhibitor	Decreases 6 mA methylation of mtDNA and inhibits inflammasome activation; attenuates mitochondrial dysfunction in atherosclerotic mice	(208)
MitoSNO	Mitochondria-targeted NO donor that limits ROS production during reperfusion	Reduces oxidative injury and apoptosis	(212)
Renal denervation (RDN)	Reduces norepinephrine and MAO-A-induced mitochondrial ROS	Alleviates endothelial inflammation and plaque burden in ApoE^−/−^ mice	(207)
Elamipretide (SS-31)	Stabilizes cardiolipin and protects mitochondrial respiration and integrity	Restores mitochondrial function and establishes homeostasis in cardiovascular disease models	(212)
Mitochondrial transplantation	Direct delivery of healthy mitochondria to macrophages	Attenuates plaque progression and promotes plaque stabilization in murine model	(213)
MTHFR	Folate cycle enzyme	Converts homocysteine to methionine, and the genetic variants increase vascular risk and mitochondrial dysfunction	(214)
Luteolin	Mitochondrial oxidative stress blocker	Inhibits mtROS-mediated inflammation, apoptosis, and EC dysfunction	(163, 164)
Thiazolidinedione and PPARα/γ agonists	PPAR modulators	Regulate mitochondrial lipid oxidation and dynamics, reduce lipid accumulation and vascular inflammation	(190)
Mdivi-1, P110	Mitochondrial dynamics modulator	Balances fusion and fission and protects against oxidative damage and mitochondrial fragmentation	(215)

## 6 Conclusion

Endothelial mitochondria function as central regulators of vascular homeostasis whose dysfunction drives atherosclerosis initiation and progression. Through integration of redox signaling, inflammatory responses, and mitochondrial quality control, endothelial mitochondria operate upstream of classical hallmarks of endothelial dysfunction. Restoring mitochondrial homeostasis represents a promising therapeutic avenue, and future studies defining selective endothelial mitochondrial targeting strategies will be critical for translating these insights into effective therapies.
